# Optimum neural tuning curves for information efficiency with rate coding and finite-time window

**DOI:** 10.3389/fncom.2015.00067

**Published:** 2015-06-03

**Authors:** Fang Han, Zhijie Wang, Hong Fan, Xiaojuan Sun

**Affiliations:** ^1^College of Information Sciences and Technology, Donghua UniversityShanghai, China; ^2^Engineering Research Center of Digitized Textile and Fashion Technology, Ministry of Education, Donghua UniversityShanghai, China; ^3^Glorious Sun School of Business and Management, Donghua UniversityShanghai, China; ^4^School of Science, Beijing University of Posts and TelecommunicationsBeijing, China

**Keywords:** neural tuning curve, information efficiency, rate coding, finite-time window, logistic function

## Abstract

An important question for neural encoding is what kind of neural systems can convey more information with less energy within a finite time coding window. This paper first proposes a finite-time neural encoding system, where the neurons in the system respond to a stimulus by a sequence of spikes that is assumed to be Poisson process and the external stimuli obey normal distribution. A method for calculating the mutual information of the finite-time neural encoding system is proposed and the definition of information efficiency is introduced. The values of the mutual information and the information efficiency obtained by using Logistic function are compared with those obtained by using other functions and it is found that Logistic function is the best one. It is further found that the parameter representing the steepness of the Logistic function has close relationship with full entropy, and that the parameter representing the translation of the function associates with the energy consumption and noise entropy tightly. The optimum parameter combinations for Logistic function to maximize the information efficiency are calculated when the stimuli and the properties of the encoding system are varied respectively. Some explanations for the results are given. The model and the method we proposed could be useful to study neural encoding system, and the optimum neural tuning curves obtained in this paper might exhibit some characteristics of a real neural system.

## Introduction

To some extent, a neural system can be viewed as an information processing system, where information from the environment is encoded by the system and then processed by another. Many neural encoding schemes are proposed, among which firing rate coding scheme has been extensively explored. Neural tuning curves, or stimulus-response curves, are often used to model the input-output relationship of neurons, where the neural coding scheme is usually rate coding. To construct such models, one needs to collect the firing rates of an isolated neuron presented by given inputs. The neuron is then treated as a “black box” and is fitted using the data with a certain function, i.e., one does not need to know the details of the underlying mechanisms of the neurons; he only needs to find a function to fit the input-output data well. This raises an important question here. That is, though these tuning curves fit the input-output data of the neurons well, why real neurons process information in such a way?

Information theory (Alexander and Frédéric, 1999; McDonnell et al., 2011; Rolls and Treves, 2011) can be used to explain the underlying mechanisms of information processing for neural systems, which may consist of only one single neuron (Ikeda and Manton, 2009) or a population of neurons (Ganguli and Simoncelli, 2014). It is suggested that the neural systems have evolved to be optimum information processing systems during the long time evolution in the rigorous environment (Mlynarski, 2014). Namely, the neural systems are optimized by the evolution process of the nature to convey more information with less energy. Three factors should be considered for an optimum neural encoding system (Kostal and Lansky, 2013). Firstly, mutual information, rather than entropy of the neural responses, should be used to quantify the information processing capacity of the neurons. This is because mutual information represents the amount of information conveyed by the neural responses about a set of stimuli, and large entropy does not mean large mutual information. Secondly, the factor of encoding time should be considered (Bethge et al., 2003a). As a matter of fact, neurons have to complete the encoding task within a short time period. This is because the subsequent stimulus might come in very short time and the neural system has to encode ceaselessly these arriving stimuli. Furthermore, to ensure a rapid response of the neural system to the stimulus, which is vital for creatures to survive in a rapidly changing environment, the encoding process must be finished in very short time. Thirdly, energy consumption needs to be included in the system (Kostal and Lansky, 2013; Biswa et al., 2014). This is rational because energy consumption of the neural system occupies a considerable portion of the total energy consumption of creatures (Zhu et al., 2002), and less energy consumption implies more chance of survival in the rigorous environment.

There have been many studies on determining the tuning curves of the neurons using information theory. The method of entropy maximization of the information theory is used to determine the tuning curves of the neurons given that the distribution of the stimuli is known (Dayan and Abbott, 2001). Neural systems are optimized based on Fisher information (McDonnell and Stocks, 2008). The tuning curves of the neural systems are optimized to get largest mutual information (Nikitin et al., 2009). The mutual information is optimized if the tuning curves of neurons are discrete and these discrete values are obtained by gradient decent method (Nikitin et al., 2009). With mutual information theory, the optimal strength of the electrical synapses is determined to achieve a least ratio of energy to information (Moujahid et al., 2011). There are also some researches concerning the analysis of optimal tuning functions (Bethge et al., 2003b; Yaeli and Meir, 2010).

However, the studies on optimum tuning curves concerning all the three aforementioned factors are insufficient. The aim of this paper is to investigate what kind of neural tuning curves could make a neural encoding system with finite-time window have a high information efficiency, i.e., can convey more information about a set of stimuli with less energy consumption. This paper is organized as follows. In Section Model and Method, the model of the neural encoding system is described; a calculation method for calculating the mutual information for stimulus with variable steps is proposed and the definition of information efficiency is introduced. In Section Results, it is shown that Logistic functions are the optimum tuning curves of the neural system by analyzing the effects of the neuronal channel noise and the energy consumption on the optimum tuning curve and by comparing the values of the information efficiency obtained by Logistic functions and other functions. The relationship between the information efficiency and the parameters of the Logistic function is investigated, and the optimum combinations of the parameters for maximizing the information efficiency are also explored. Conclusions and discussions are presented in Section 4.

## Model and method

In this section, a finite-time neural encoding system based on the firing rate coding is presented. A method for calculating the mutual information of the encoding system is proposed.

### Model neural system with poisson neurons

Stimuli are inputted into a neuron (or a population of neurons), which encodes the stimuli into the firing rates. The strength of the stimuli (e.g., the light intensity) is supposed to be continuous and obeys Gaussian distribution, of which the probability density is described by:

(1) $$\text{p}\left(\text{s}\right)=\frac{1}{\sqrt{\text{2}\pi }\sigma }\text{exp}(-\frac{{\left(\text{s}-\bar{\text{s}}\right)}^{2}}{2\sigma^{2}})$$

where s is the strength of the stimulus, sϵ[s_min_, s_max_]; s is the mean of the stimulus strength.

The spike sequence is assumed to be Poisson process, as the neural responses are usually noisy and often modeled by Poisson statistics (Dayan and Abbott, 2001; Nikitin et al., 2009). Suppose y = λ (*s*) is the firing rate (response) function of the neuron, then for every stimulus s, the neuron will output Poisson spike sequences with the mean firing rate λ (*s*). We assume the encoding task be completed within a short time window T.

### Mutual information

We use mutual information to characterize the amount of stimulus information encoded in the number of spikes emitted by the neuron. Let H be the full response entropy, which is described by

(2) $$\text{H}=\sum_{r\;=\;0}^{\infty }{\text{p}}_{\text{r}}{\text{log}}_{2}({\text{p}}_{\text{r}})$$

where r = 0, 1, 2, …, ∞ is the number of spikes of the neuron; p_r_ is the probability of a response r and is related to the conditional probability p(r|s) and the probability density p(*s*) that stimulus s is presented as follows

(3) $${\text{p}}_{\text{r}}=\int_{{\text{s}}_{\text{min}}}^{{\text{s}}_{\text{max}}}\text{p}\left(\text{r}|\text{s}\right)\text{p}\left(\text{s}\right)\text{ds}$$

Let H_n_ be the noise entropy which is caused by the noisy nature of the neural response, which is calculated by

(4) $${\text{H}}_{\text{n}}=\sum_{\text{r}}\int_{{\text{s}}_{\text{min}}}^{{\text{s}}_{\text{max}}}\text{p}\left(\text{s}\right)\text{p}(\text{r}|\text{s}){\text{log}}_{2}(\text{p}\left(\text{r}|\text{s}\right))\text{ds}$$

with

(5) $$\text{p}\left(\text{r}|\text{s}\right)=\frac{{\left(\lambda \left(\text{s}\right)\text{T}\right)}^{\text{r}}{\text{e}}^{-(\lambda \left(\text{s}\right)\text{T})}}{r!}$$

(6) $$\lambda \left(\text{s}\right)={\text{F}}_{\text{max}}\text{f}(\text{s})$$

where p(r|s) is the conditional probability; λ (s) is the mean firing rate (response) of the neuron corresponding to the stimulus strength s, representing the tuning curve of the neuron; f(s) is normalized tuning curve of the neuron; and F_max_ is the maximum firing rate of the neuron.

Then the mutual information can be obtained by

(7) $${\text{I}}_{\text{m}}=\text{H}-{\text{H}}_{\text{n}}.$$

According to Equation (6), λ (s)T is the average number of spikes within time window T. In terms of Equations (3) and (5), p(r|s) and p(r) keep unchanged if F_max_ T keeps invariant (note that λ (s)T = F_max_Tf(s)). Thereby H, H_n_, and I_m_ keep unchanged if F_max_ T keeps invariant. Consider that a population of N neurons, where neuron *i* has a maximum firing rate F^i^_max_, is used to encode the stimulus. The N neurons are assumed to receive the same stimulus but response to the stimulus statistically independently. The number of spikes emitted by neuron *i*, n_i_, within the time window T, is a random variable of Poisson distribution with mean F^i^_max_ f(s)T and variance F^i^_max_ f(s)T. As the N neurons in the population respond to the stimulus independently, the total number emitted by the N neurons within the time period T, r = ∑_i_ n_i_ is also a random variable of Possion distribution with mean ∑_i_ F^i^_max_ f(s)T and variance ∑_i_ F^i^_max_ f(s)T. Therefore, the population encoding system with N neurons is equivalent to a one-neuron system with maximum firing rate being the summation of the N maximum firing rates of the N-neuron system, i.e. F_max_ = ∑_i_ F^i^_max_ f(s). Therefore, we only discuss one-neuron system and treat F_max_ T as one parameter in the following analysis. Finite-time window means that T is not very large, implying that F_max_ T is not very large if the population size of the neurons is not very large either.

### Information efficiency

Since spike generation and transmission occupy the main part of the energy consumption in the brain (Zhu et al., 2002; Kostal and Lansky, 2013), we use the number of spikes to represent the energy consumption for the encoding system. Thereby the energy consumption, E, can be described by E = ∑_r_ p(r)r. We use an objective function that takes account of both the mutual information and the energy consumption to characterize the information encoding efficiency (denoted by I_E_), which is written as

(8) $${\text{I}}_{\text{E}}=(2^{{\text{I}}_{\text{m}}}-1)-\gamma E.$$

We determine the value of the parameter γ by defining that the value of the objective function should be zero if the neuron does “nothing” but just amplifies the input signals by a factor of TF_max_/(s_max_ − s_min_) and lets them pass through. For example, given a neural encoding system with s_min_ = − 2, s_max_ = 2, σ = 1, and TF_max_ = 300, we set the tuning curve of the neuron as (s + s_min_)^*^ TF_max_/(s_max_ − s_min_). This linear tuning curve does “nothing” except for amplifying the stimulus and taking a translation. For the given parameters s_min_, s_max_ and σ, a sole γ could be determined. Here, we get I_m_ = 2.2177 and *E* = 150 by numerical simulations, and hence we can get γ = 0.0233. γ is set at 0.0233 unless otherwise stated in this paper. Therefore, if a tuning curve is better than the linear tuning curves, the information efficiency will be larger than zero; otherwise, it will be less than zero. The better the tuning curve is, the larger value of information efficiency will be.

### Calculations with variable sampling step

To calculate the mutual information, we sample the stimulus strength into discrete points as s_i_, i = 1, 2, 3, … M. The stimulus strength of s_i_ corresponds to the firing rates F_max_ f(s_i_). The response of the neuron will be a random variable obeys Poisson distribution with mean r = F_max_ Tf(s_i_) and variance F_max_ Tf(s_i_). The conditional probability of the discrete version p(r_j_|*s*_i_) will be calculated as $\frac{{\left(\lambda \left(s_{\text{i}}\right)\text{T}\right)}^{\text{r}}{\text{e}}^{-\left(\lambda \left(s_{\text{i}}\right)\text{T}\right)}}{{\text{r}}_{\text{j}}!}$, and $\text{p}(s_{\text{i}})=\frac{1}{\sqrt{2\pi }\sigma }\text{exp}\epsilon (-\frac{{\left({\text{s}}_{\text{i}}-\bar{\text{s}}\right)}^{2}}{2\sigma^{2}})(s_{\text{i}+1}-s_{\text{i}})$. According to Equation (3), we have to carry out 3Mceil(F_max_ Tf(s_i_)) times of multiplication to obtain H_*n*_. Therefore, if M and F_max_ Tf(s_i_) are large (note that F_max_ T may be the summation of the N maximum firing rates of the N-neuron system, thereby F_max_ Tf(s_i_) may be large if the population size is large), large amount of calculations is needed, especially when we search for the optimum parameter combinations to maximize I_E_ (in this case, we need to calculate I_E_ under different values of parameter combinations). To reduce the calculation burden, we propose a sampling scheme with variable step size in this paper. We sample f(s) into discrete points f(s_j_), j = 0, 1, 2, …, M. Δf(s_j_) = f(s_j_) − f(s_j − 1_). Δf(s_j_) can be different from Δf(s_i_) for i ≠ j, which is determined by follows.

As H_n_ = ∑_i, j_ p(s_i_) p(r_j_|*s*_i_) log^p (r_j_|s_i_)^ and H = ∑_j_ p(r_j_) log^p (r_j_)^ (r_j_ represents the neuronal response of *j* spikes), we can see that if r_j_ ≫ F_max_ Tmax(f(s_i_)), then p(r_j_|*s*_i_) ≈ 0 and p(r_j_) ≈ 0. Therefore, we limit the range of r as 0 < r < [2maxϵ(f(s_i_))] in this paper ([.] means getting the integer part of the number). Furthermore, as the larger F_max_ Tf(s_i_) is, the closer the neighboring conditional probabilities become (For example, if F_max_ Tf(s_i_) is very large, p(*r*_j − 1_|*s*_i_) ≈ p(*r*_j_|*s*_i_) ≈ p(*r*_j + 1_|*s*_i_)). Based on this observation, we propose a sampling scheme with variable step size. When F_max_ Tf(s_i_) is small (f(s_i_) < 1 in this paper), we let Δf(s_j_) = f(s_j_) − f(s_j − 1_) = h/F_max_ T. *h* is 0.001 in this paper. When F_max_ Tf(s_i_) ≥ 1, we let. $△f\left(s_{\text{j}}\right)=\text{h}\sqrt{{\text{F}}_{\text{max}}\text{Tf}({\text{s}}_{\text{i}})}/{\text{F}}_{\text{max}}\text{T}$ According to this sampling scheme, we can get discrete points f(s_j_), j = 0, 1, 2, …, R. Accordingly, we obtain the discrete points of the stimuli s_j_ which produce f(s_j_). Thus, the continuous variable of the stimuli, s, is discretized. Owing to the sampling scheme with variable step size and the limitation of the range of the value of *r*_j_, the computational efficiency is greatly improved.

## Results

### Optimum neural tuning curves based on information efficiency

**Figure 1:**
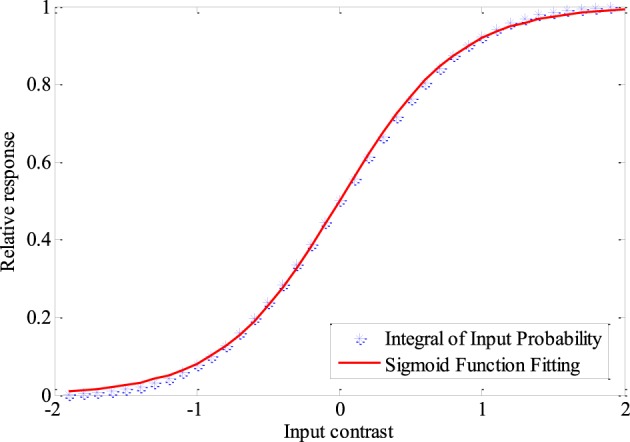
**The integral of the probability density of the stimuli and the fitted Logistic function**. s_min_ = − 2, s_max_ = 2, σ = 1, s = 0. The steepness of the logistic function is 0.41. Fixed sampling step is adopted to show the integration process clearly in this Figure.

A very important question is what kind of tuning curves are the optimum tuning curves for the neural coding system. The expected shape of the neural response distribution when there is no noise in the neuronal channel can be obtained easily, basing on the fact that neurons with tuning curves resulting from entropy maximization have maximum mutual information. It is known that the tuning curves corresponding to the integral of the probability density of the stimulus (see Figure 1) leads to histogram equalization of the neural response and thus results in maximum entropy. Such tuning curves can be fitted quite well by Logistic functions when the stimulus follows norm distribution, which is expressed as

(9) $$f\left(s\right)=1/(1+e^{\frac{s}{\epsilon }}),$$

where ϵ represents the steepness of the function.

When the neuron channel is noisy, maximum entropy cannot lead to maximum mutual information, i.e., histogram equalization of the neuronal responses (each neuronal response has the same probability) cannot lead to maximum mutual information necessarily. Furthermore, histogram equalization cannot lead to least energy consumption as well. Then, what is the optimum tuning curve if both of noise and energy consumption are considered?

If the noisy channel of neurons is Gaussian and independent of the inputs, then maximum entropy leads to maximum mutual information if energy consumption is neglected. The estimate for λ (s), λ_est_ = r/T, will be a Gaussian variable with mean λ and variance λ /T. Its square root will have mean $\sqrt{\lambda }$ and variance 1/(2T). As the variance of $\sqrt{\lambda_{\text{est}}}$ is independent of, λ, $\sqrt{\lambda }$ should have the maximum entropy distribution. This means that if the response of the neuron obeys Poisson distribution, the optimum tuning curves would have relationship with the function ${\left(\frac{1}{1+e^{\frac{s}{\varepsilon }}}\right)}^{2}$ (Note that Poisson distribution approximate Gaussian distribution well when λ is large). This relationship can also be explained in the respect of noise entropy. The noise entropy described in Equation (4) can be rewritten as H_n_ = ∑_i_ H_n_ (s_i_) with ${\text{H}}_{\text{n}}\left({\text{s}}_{\text{i}}\right)=\sum_{\text{j}}\text{p}\left({\text{s}}_{\text{i}}\right)\text{p}\left({\text{r}}_{\text{j}}|{\text{s}}_{\text{i}}\right){\text{log}}^{\text{p}\left({\text{r}}_{\text{j}}|{\text{s}}_{\text{i}}\right)}=\sum_{\text{j}}\text{p}\left({\text{s}}_{\text{i}}\right)\text{p}\left({\text{r}}_{\text{j}}|\bar{\text{r}}\right){\text{log}}^{\text{p}\left({\text{r}}_{\text{j}}|\bar{\text{r}}\right)}$ where r = Tλ (s). Since p(r_j_|r) obeys possoin distribution with variance r, small values of λ (s) leads to small value of H_n_ (s_i_). Therefore, ${\left(\frac{1}{1+{\text{e}}^{\frac{\text{s}}{\varepsilon }}}\right)}^{2}$ or $\frac{1}{1+{\text{e}}^{\frac{\text{s}-\mu^{\prime }}{\epsilon }}}$ with μ′ > 0 will be a better tuning curve for reducing noise entropy than $\frac{1}{1+{\text{e}}^{\frac{\text{s}}{\epsilon }}}$. Adding the energy cost (determined by the parameter γ) will also tend to push λ (s) to lower values, since lower firing rate results in lower energy consumption. Therefore, considering the three factors, i.e., the integral of input probability distribution, noise entropy, and the energy consumption all together, the optimum tuning curve may take a form like $\left[{\frac{1}{1+{\text{e}}^{\frac{\text{s}-\mu^{\prime }}{\epsilon }}}}^{\theta }\right]$ with θ >1 *and* μ′ > 0. As a matter of fact, for any input probability distribution, the curve of the integral of the input probability (suppose that it is fitted by f(s)) is the optimum tuning curve if noise is neglected and γ = 0; f(s − μ)^θ^ may be the optimum tuning curve if both noise and energy are considered.

**Figure 2:**
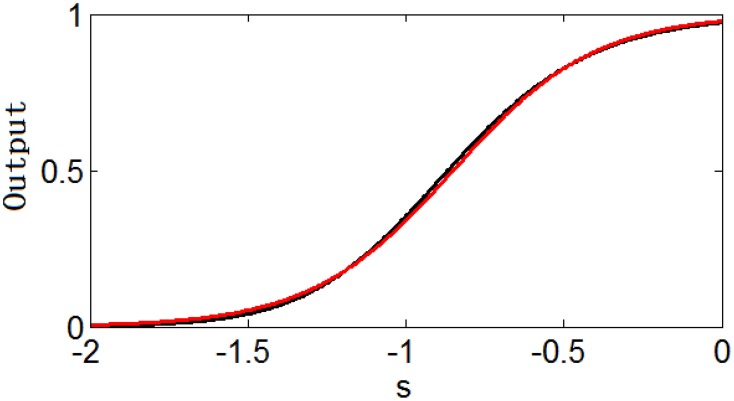
**Similarity of the function $\left[{\frac{1}{1+{\text{e}}^{\frac{\text{s}}{0.5}}}}^{1.5}\right]$ (black) and the function $\frac{1}{1+{\text{e}}^{\frac{\text{s}-0.3}{0.45}}}$ (red)**.

However, it is interesting that ${\left[\frac{1}{1+{\text{e}}^{\frac{\text{s}}{\epsilon }}}\right]}^{\theta }$ or ${\left[\frac{1}{1+{\text{e}}^{\frac{\text{s}-\mu^{\prime }}{\epsilon }}}\right]}^{\theta }$ can be approximated well by the simple logistic function $\frac{1}{1+{\text{e}}^{\frac{\text{s}-\mu }{\varepsilon }}}$ with the parameter values of ε and μ (the translation of the function) determined by the parameters θ and ϵ (Note that ${\left[\frac{1}{1+{\text{e}}^{\frac{\text{s}-\mu^{\prime }}{\epsilon }}}\right]}^{\theta }$ can be changed to ${\left[\frac{1}{1+{\text{e}}^{\frac{\text{s}}{\epsilon }}}\right]}^{\theta }$ by replacing s − μ′ with s′ = s − μ′). Figure 2 shows an example of the similarity of the function ${\left[\frac{1}{1+{\text{e}}^{\frac{\text{s}}{\epsilon }}}\right]}^{\theta }$ and the function $\frac{1}{1+{\text{e}}^{\frac{\text{s}-\mu }{\varepsilon }}}$. Table 1 shows the parameter projection of the function ${\left[\frac{1}{1+{\text{e}}^{\frac{\text{s}}{\epsilon }}}\right]}^{\theta }$ to the function $\frac{1}{1+{\text{e}}^{\frac{\text{s}-\mu }{\varepsilon }}}$. This means that the family of the logistic functions are the tuning curves that are as good as (or might even better than) the family of the functions ${\left[\frac{1}{1+{\text{e}}^{\frac{\text{s}-\mu^{\prime }}{\epsilon }}}\right]}^{\theta }$. Therefore, we assume that the optimum tuning curve for a noisy system when energy consumption is considered can be described by $\frac{1}{1+{\text{e}}^{\frac{\text{s}-\mu }{\varepsilon }}}$. From the above discussion, it is conceivable that the optimum parameter values of this tuning curve depend on p(s), γ, F_max_, and T, which we will discuss in the next section.

**Table 1:** **functions $\left[{\frac{1}{1+{\text{e}}^{\frac{\text{s}}{\epsilon }}}}^{\theta }\right]$ approximated by functions $\frac{1}{1+{\text{e}}^{\frac{\text{s}-\mu }{\varepsilon }}}$**.

${\left[\frac{1}{1+{\text{e}}^{\frac{\text{s}}{\epsilon }}}\right]}^{\theta }$	θ	1.5	1.5	1.5	2	2	2	2.5	2.5	2.5
	ϵ	0.1	0.3	0.5	0.1	0.3	0.5	0.1	0.3	0.5
$\frac{1}{1+{\text{e}}^{\frac{\text{s}-\mu }{\varepsilon }}}$	ε	0.09	0.27	0.45	0.08	0.25	0.42	0.07	0.23	0.40
	μ	0.05	0.15	0.3	0.1	0.3	0.5	0.15	0.4	0.6

To check whether the Logistic function is the best tuning curve for the information efficiency, other types of tuning curves are also adopted and comparisons are taken. Let's considering power functions f(s) = α + *s*^β^, which are commonly used forms for tuning curves (Poirazi et al., 2003). We make a minor modification on them to adapt to our model as follows:

(10) $$\text{f}\left(\text{s}\right)=\frac{\alpha +{\left(s+s_{min}\right)}^{\beta }}{\alpha +{\left(s+s_{max}\right)}^{\beta }}{\text{TF}}_{\text{max}}.$$

**Figure 3:**
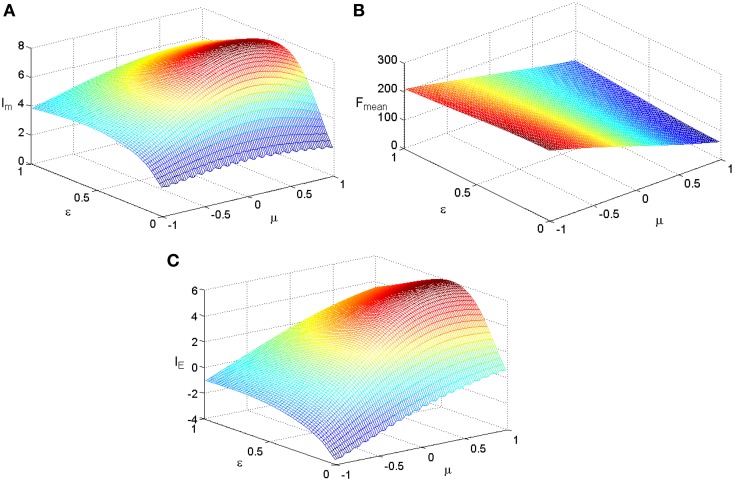
**The dependency of mutual information, energy consumption, and information efficiency on the two parameters of the Logistic function**. **(A)** Mutual information. **(B)** Energy consumption. **(C)** Information efficiency.TF_max_ = 300, other parameter values are set the same as those in Figure 1.

**Figure 4:**
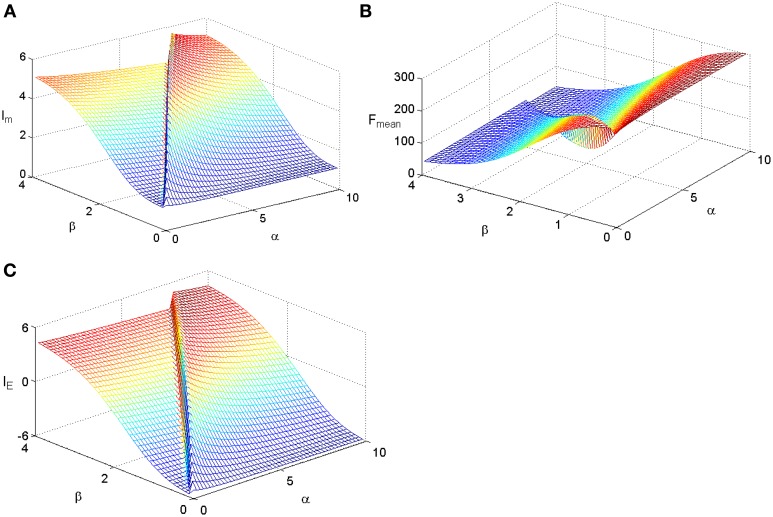
**The dependency of mutual information, energy consumption, and information efficiency on the two parameters of the power function**. **(A)** Mutual information. **(B)** Energy consumption. **(C)** Information efficiency. The parameter values are set the same as those in Figure 3.

We fix TF_max_ = 300 and stimuli variance σ = 1 and carry out simulations for the two kinds of tuning curves. The simulations cover the whole parameter space spanned by ε and μ and the results are plotted in Figure 3 for the Logistic function. The simulations for the tuning curves of power functions are also carried out. We searched the whole space spanned by α and β, and the dependency of mutual information, energy consumption, and information efficiency on the two parameters are shown in Figure 4. The plateaus of (Figures 3, 4) represent the regions where the parameters of the tuning curves are set at appropriate values, i.e., these parameter values lead to high mutual information or high I_E_. Therefore, it is rational that we compare the height of the two plateaus to identify the better tuning curves for information efficiency. It can be seen that the plateau of information efficiency resulting from the Logistic function is higher than 6, while the one resulting from power function (Equation 10) is lower than 5. Therefore, we conclude that the Logistic function is a better tuning curve for information efficiency. Simulations with other kinds of tuning curves, exponential functions and polynomial functions, are also carried out (results not shown), and the Logistic function is better than these functions as well. We also carried out simulations when stimuli variance σ is varied. The information efficiencies corresponding to various stimuli distributions for Logistic functions are also higher than those found by other kinds of functions (the results not shown in this paper). Therefore, we can conclude that Logistic functions are the best tuning curves for information efficiency.

### Relationship between information efficiency and the parameters of tuning curves

**Figure 5:**
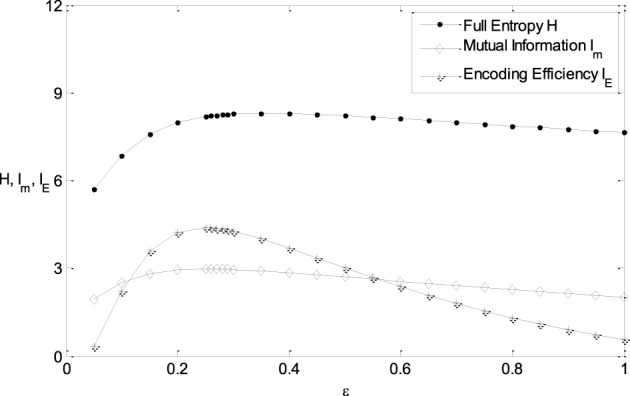
**The relationship between ε and the full entropy, mutual information, and the information efficiency**. μ = 0; other parameter values are set the same as those in Figure 3.

It is clearly shown in Figure 3B that the energy consumption is sensitive to the parameter μ and insensitive to the parameter ε of the Logistic function. The energy consumption decreases with the increasing of μ. It can be further seen from Figure 3 that the mutual information and I_E_ increase with the increasing of one of the parameter (ε or μ), reach the peak, and then decrease, if the other parameter is fixed. To reveal the relationship between the information efficiency and the two parameters more clearly, we first fix one parameter and then vary the other parameter. Figure 5 shows the relationship between ε and the full entropy, mutual information, and the information efficiency, and it is shown that the full entropy is very low when ε is very small. That is because when ε is very small, the Logistic function is very steep, and therefore the too steep Logistic function is far from the curve plotted by the integral of the probability density (see Figure 1), resulting in small value of full entropy. With the increasing of ε, the Logistic function gets closer to the integral of the probability density, resulting in higher full entropy. The highest full entropy occurs at about ε = 0.3 (It is worthy of noting that totally overlap of the Logistic function and the integral of the probability function may not result in the highest full entropy due to the noisy nature of the neuron) and then decreases with the further increasing of ε. It is very interesting to see that the shape of the curve of the mutual information is almost the same as that of the full entropy. Additionally, the energy consumption keeps approximately constant due to the invariance of the parameter μ (see Figure 3B). Therefore, the dependency of I_E_ on parameter ε (see Figure 5B) is approximately the same as that of I_m_ (It is worthy of noting that I_E_ is not measured in unit of bit as I_m_, and the negative value of I_E_ means that the corresponding tuning curve is even worse than the one just linearly amplifying the stimuli). In short, if μ is fixed, the full entropy is sensitive to the parameter ε The curves for the full entropy, mutual information and the information efficiency have the same shape.

**Figure 6:**
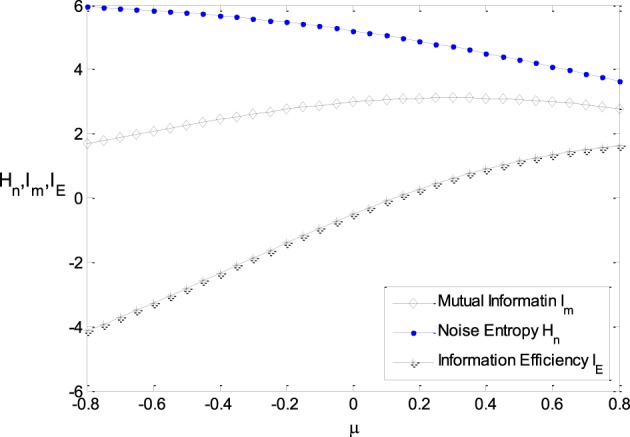
**The relationship between μ and the noise entropy, mutual information, and the information efficiency**. ε = 0.25, other parameter values are set the same as those in Figure 3.

Figure 6 shows the relationship between μ and the noise entropy, mutual information and the information efficiency. It can be seen from Figure 6 that the noise entropy is sensitive to the parameter μ. It decreases with the increasing of μ, resulting in the increasing of mutual information when μ is not too large. It can be seen from Figure 3B clearly that the energy consumption decreases with increasing μ rapidly. Thereby, the information efficiency increases with increasing μ rapidly as well. Therefore, a Logistic function with its location being shifted a little to the right side of the horizontal axis has higher information efficiency.

### Optimum parameter combinations for various stimuli distribution and coding window

**Figure 7:**
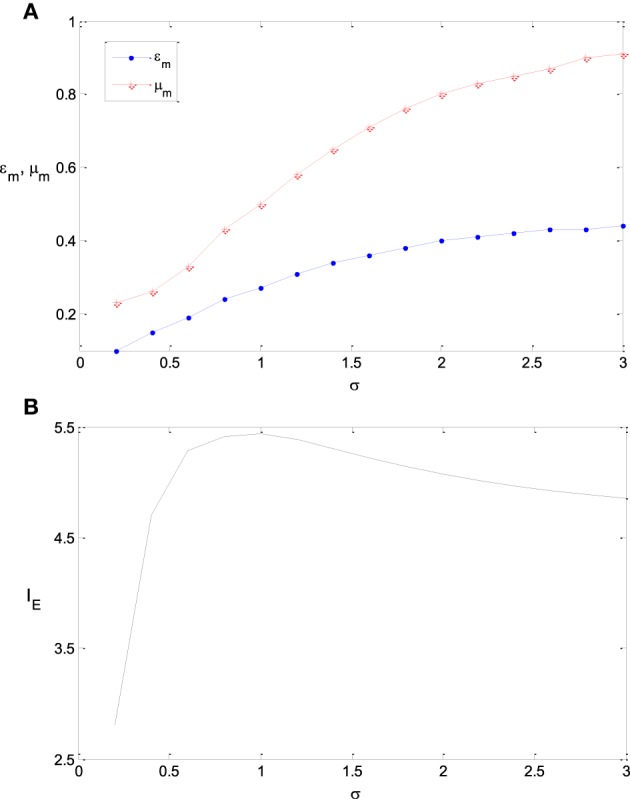
**The dependency of ε_m_ and μ_m_ on σ and the corresponding maximum information efficiency**. **(A)** The dependency of ε_m_ and μ_*m*_ on σ; **(B)** The corresponding maximum information efficiency. The parameter values are set the same as those in Figure 3.

**Figure 8:**
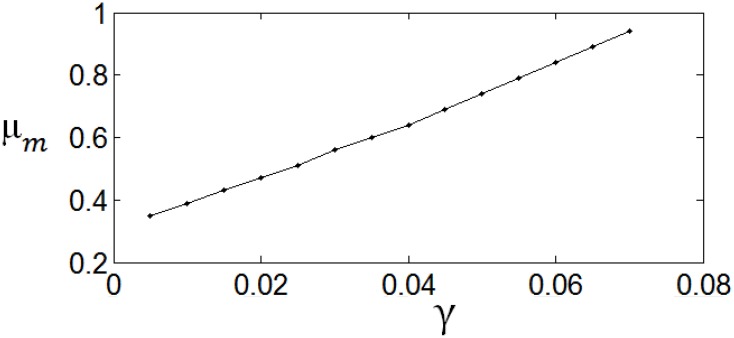
**The dependency of μ_*m*_ on γ. The parameter values are set the same as those in Figure 3**.

**Figure 9:**
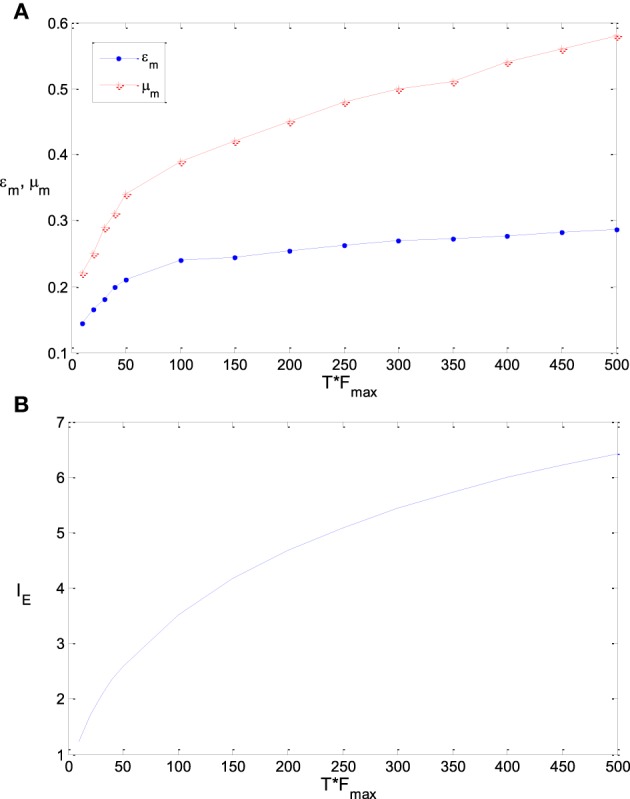
**The dependency of ε_m_ and μ**_***m***_ **on TF_max_ and the corresponding maximum information efficiency**. **(A)** The dependency of ε_m_ and μ_*m*_ on TF_max_; **(B)** The corresponding maximum information efficiency. The parameter values are set the same as those in Figure 3.

We further explore the optimum parameter combinations of ε_m_ and μ_m_ to maximize the information efficiency. As discussed in the first subsection of this section, it is intuitive that the optimum tuning curve is the curve of the integral of the input probability if T is very large and γ = 0. Thereby ε_m_ is the steepness of the logistic function that fits this integral curve, and μ_m_ = 0. Therefore, ε_m_ will be large if the input probability distribution is flat (i.e., σ is large), while ε_m_ will be small if σ is small. On the other hand, if T is small and γ is large, then functions of ${\left[\frac{1}{1+{\text{e}}^{\frac{\text{s}-\mu^{\prime }}{\epsilon }}}\right]}^{\theta }$ with large values of θ and μ′ may be good tuning curves. Correspondingly, the logistic function $\frac{1}{1+{\text{e}}^{\frac{\text{s}-\mu }{\varepsilon }}}$ with small value of ε and large value of μ may be good tuning curves (see the parameter mapping of the two families of functions in Table 1). This implies that ε_m_ will be small and μ_m_ will be large if T is small and γ is large. These intuitive observations are confirmed by simulation results shown by Figures 7–**9**.

Figures 7A,B shows the dependency of ε_m_ and μ_m_ on the parameter σ and the corresponding maximum information efficiency. It is shown that with the increase of the variance of stimulus distribution σ, the optimum parameter value of ε_m_ increases monotonously. The maximum information efficiency is low when σ is small (see Figure 7). This is because the entropy of the input signal is low in the case of low value of σ. The maximum information efficiency increases with σ and reaches the peak value 5.4 when σ = 1.

Figure 8 shows the dependency of μ_m_ on the parameter γ. It is shown that if γ is large, i.e., the energy consumption is heavily weighted, then μ_m_ is large. Namely, μ_m_ increases with the increase of. γ. ε_m_ is insensitive to the parameter value of γ.

Figure 9A shows the sensitivity of ε_m_ and μ_m_ to the parameter T, namely TF_max_ (see the explanation of this joint parameter in Section Model and Method). It is found that the optimum parameter values of ε_m_ and μ_m_ increase if TF_max_ gets increased. As the variance of λ_est_, λ (s)/T, approaches to 0 when T is very large, ε_m_ will be exactly the steepness of the Logistic function that fits the integral of input probability (around 0.41 according to Figure 1), if the energy consumption is neglected. As discussed previously, ε_m_ will be much smaller than 0.41 when T is very small due to the noise effect. Therefore, the value of ε_m_ increases with the increasing of TF_max_. Energy consumption is very sensitive to the parameter T. It is proportional to T if other parameters keep invariant. To save the energy thereby to increase the information efficiency, μ_m_ needs to be increased with the increasing of T (Note that μ_m_ is very sensitive to the energy consumption according to Figure 8). This explains the plot of μ_m_ vs. TF_max_ in Figure 9A. If TF_max_ gets larger, the according maximum information efficiency also gets larger (see Figure 9B).

## Conclusions and discussions

We use information theory to search the optimum neural tuning curves to maximize the information efficiency. The information efficiency considered in this paper concerns three factors, i.e., mutual information, coding time window and energy consumption.

We proposed a finite-time neural encoding system, where the spike sequence of the neuron corresponding to a stimulus obeys Poisson process and the external stimuli obey norm distribution. We also propose a calculation method based on the variable sampling step to calculate the mutual information and the information efficiency. The effects of the neuronal channel noise and the energy consumption on the optimum tuning curve are analyzed and the calculations of the mutual information and the information efficiency are carried out. It is found that the Logistic functions are the best tuning curves in the sense that the information efficiency resulting from Logistic functions is higher than that resulting from other kinds of functions. Then we study the relationship of the information and information efficiency of the neural system with the parameters of Logistic tuning curves. It is revealed that the parameters representing the steepness of the Logistic function (ε) relates more closely with the full entropy, while the parameters representing the location of the function in the horizontal axis (μ) relates more closely with the noise entropy and energy consumption. The curves for the full entropy, mutual information and the information efficiency have the same shape if the parameter representing the location is fixed, while a Logistic function with its location being shifted a little to the right side of the horizontal axis has higher information efficiency if the parameter ε is fixed. We further explore the optimum combinations of the parameter values of the Logistic tuning curve for maximizing the information efficiency when the properties of the stimuli and the neural system vary. It is shown that with the increase of the variance of stimulus distribution, the optimum parameter value of parameter representing the steepness (ε_m_) increases monotonously; ε_m_ increases when the encoding time window or maximum firing rate of the neuron gets larger; and μ_m_ increases with the increase of γ. Our results consist with the fact that Logistic functions, which could fit experimental data very well in many neural experiments, may be the actual tuning curves in many real neural systems (Dayan and Abbott, 2001; Poirazi et al., 2003; McDonnell and Stocks, 2008). And also, the results about the optimum parameters of the Logistic function might be some characteristics of a real neuronal information processing system.

**Figure 10:**
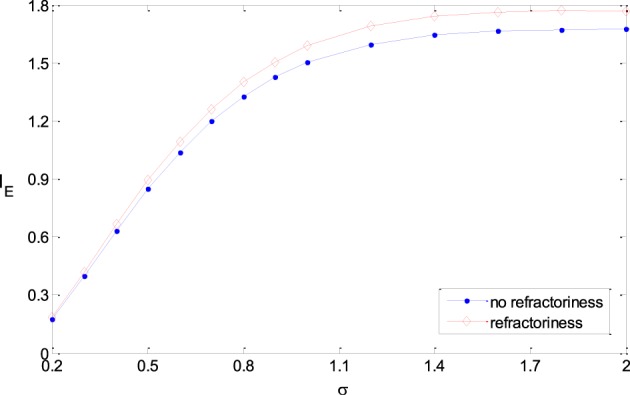
**Comparison of the information efficiency of the simple neural model and the model with refractoriness under the different parameters**. The refractoriness is 5 ms, TF_max_ = 20, ε = 0.25, μ = 0.5, σ varies from 0.2 to 2.

In this paper, we used Poisson process to model the output of noisy rate-coding neurons. The result in this paper can be extended to more real neural models, for example, Poisson process with absolute refractoriness (Dayan and Abbott, 2001). Poisson process with absolute refractoriness means that a neuron cannot fire until the fixed time period due to the refractoriness is finished. After the refractoriness, the spike intervals of the firing sequences follow exponential distribution. The simulation results show that all the results got in the paper are valid when the refractoriness is much less than the mean of the spike intervals, which is actually the case in real neural systems. Figure 10 shows that the information efficiency in a neural model with refractoriness is roughly equal to (or a slighter higher than) that of Poisson process model (The two neural models have the same inputs and the same tuning curves). Therefore, the results got in previous sections with Poisson process neural models are valid for the more real neural models.

### Conflict of interest statement

The authors declare that the research was conducted in the absence of any commercial or financial relationships that could be construed as a potential conflict of interest.
